# Construction Notes

**Published:** 1896-06-06

**Authors:** 


### CONSTRUCTION NOTES.

**Walsall Workhouse Infirmary.**

The new infirmary recently added to the buildings of the Walsall Union Workhouse has now been formally opened. The building is divided into blocks or pavilions two storeys high. Each pavilion—for males and females respectively—contains two wards 16 ft. by 13 ft. for two beds each. Each of these wards is provided with a nurse's room or kitchem 13 ft. by 13 ft., and the apartment is so arranged that from a certain window a complete supervision can be obtained of the entire ward. In this block a day-room is provided, the entrance of which is from the main corridor. The large wards are heated by Shoreland's stoves. Every means of escape, in case of fire, is also provided through an outside staircase which gives off a balcony running along the principal wards. The ventilation is through inlet wall panels, with outlet ventilators from the ceiling. Lavatories, water closets, and baths are connected with each ward. The administrative block, with accommodation for doctor and nurses, is the central block between the two ward pavilions, and it consists of dispensary, store-rooms, surgery, eight lying-in wards and four bed-rooms, separate bath-rooms, lavatories, &c. This block is heated with radiators on the low-pressure system, which is in connection with the steam from the present boilers. The outside walls of the buildings are faced with red bricks, while the window-sills and heads and other stone dressings are of Hollington stone; adamant cement plasters the walls and ceilings. The staircases are all of stone. The contractor for the work was Mr. A. Lyne; Mr. H. E. Lavender, of Walsall, being the architect; the cost of the works was about £7,000.

### New Infirmary, Lancaster.

This institution has been erected from the designs of Messrs. Austin and Paley, architects, of Lancaster. The building is well situated, and has been built at a cost of £28,000. This will give accommodation for sixty patients, but the administrative block has been completed with the view to the addition eventually of another wing. On the north side is the dispensary, on the south side the adult and the children's ward. The centre contains the administrative premises. A waiting-room adjoins the dispensary capable of accommodating eighty people; also there is here a surgeon's consulting-room, ophthalmic-room, and porter's-room. An accident corridor, 80 ft. long, leads to the main corridor. The operating-room, branching from the main corridor and approached through an ante-room, has a north light and every convenience of electric lighting. Shanks's patent antiseptic sinks are provided, and throughout the whole institution all the corners are carefully rounded off. Ten beds are provided in the children's ward. The men's and women's wards are connected with a hoist, having Bostwick's folding gates. Each ward will contain twenty-five beds. Each ward also has kitchens and nurses' rooms. A hydraulic hoist for raising patients is designed and executed by Messrs. Clark, Bunnett, and Co., of London. A large day-room overlooks Springfield Park. The administrative block contains doctor's sitting-room, matron's sitting-room, nurses' day and dining rooms, bedrooms, and stores, and a fine board-room. Every improved sanitary arrangement is to be found in the laundry and kitchen offices. There is a boiler house containing two of Stevenson and Co.'s boilers, and the steam heaters are Seward and Walton's patent.

### Melksham Cottage Hospital.

The beautifully-equipped cottage hospital, which, through the munificence of Mr. H. G. White, of Whitley, has been presented to the town, has now been formally opened. The building is approached by a carriage-way which will be bordered by trees and shrubs. The front is built of Atworth stone, rock-faced, with Westwood stone dressings. The three large mullioned windows on the ground floor are faced with plate-glass. The front door opens upon a spacious entrance hall, from which direct access can be obtained to any part of the building. On the right-hand side are the matron's sitting and bed-rooms, and on the opposite side a committee-room. A doorway in the hall communicates directly with a female ward 20 feet by 18 feet, lofty and well lighted. Connected with this is a good bath-room. A door from this ward also communicates with the corridor, which runs from the hall to the rear of the building; on the other side are the kitchen, scullery, and other offices. The surgical room, which is at the extreme end, is fitted up with all the latest appliances and instruments, the cost of which was about £100. The men's ward adjoins that of the females, and access can be gained to it either by a separate side entrance or from the corridor. This ward is the same size as the other, and has also a bath-room attached to it. The walls are covered with "Serapite," a plaster closely resembling cement. The mortuary is entirely separate from the main building. There are five bed-rooms upstairs for the officers, and they are all built over the hall, so there will be no noise from the wards. Electric bells are placed over the heads of each patient's bed. The architect was Mr. Gunstone, and the contractors Messrs. Bigwood and Co., who estimate the work at about £1,000.

